# Tumor biomechanics as a novel imaging biomarker to assess response to immunotherapy in a murine glioma model

**DOI:** 10.1038/s41598-024-66519-7

**Published:** 2024-07-06

**Authors:** Yannik Streibel, Michael O. Breckwoldt, Jessica Hunger, Chenchen Pan, Manuel Fischer, Verena Turco, Berin Boztepe, Hannah Fels-Palesandro, Jonas G. Scheck, Volker Sturm, Kianush Karimian-Jazi, Dennis A. Agardy, Giacomo Annio, Rami Mustapha, Shreya S. Soni, Abdulrahman Alasa, Ina Weidenfeld, Christopher B. Rodell, Wolfgang Wick, Sabine Heiland, Frank Winkler, Michael Platten, Martin Bendszus, Ralph Sinkus, Katharina Schregel

**Affiliations:** 1 Department of Neuroradiology, Heidelberg University Hospital, Im Neuenheimer Feld 400, 69120 Heidelberg, Germany; 2 Clinical Cooperation Unit Neuroimmunology and Brain Tumor Immunology, German Cancer Consortium (DTK) within the German Cancer Research Center (DKFZ), Heidelberg, Germany; 3 Faculty of Biosciences, Heidelberg University, Heidelberg, Germany; 4 Clinical Cooperation Unit Neurooncology, German Cancer Consortium (DTK) within the German Cancer Research Center (DKFZ), Heidelberg, Germany; 5 Department of Neurology, Heidelberg University Hospital, Heidelberg, Germany; 6 Department of Medical Oncology, Heidelberg University Hospital, National Center for Tumor Diseases, Heidelberg, Germany; 7 Department of Neurology, Medical Faculty Mannheim, MCTN, Heidelberg University, Mannheim, Germany; 8 INSERM UMRS1148-Laboratory for Vascular Translational Science, University Paris, Paris, France; 9 School of Biomedical Engineering and Imaging Sciences, King’s College London, London, UK; 10 Richard Dimbleby Laboratory of Cancer Research, School of Cancer & Pharmaceutical Sciences, King’s College London, London, UK; 11 School of Biomedical Engineering, Science and Health Systems, Drexel University, Philadelphia, USA

**Keywords:** MR elastography, Glioma, Immunotherapy, Tumor stiffness, Tissue biomechanics, Cancer imaging, Cancer models, Cancer in the nervous system

## Abstract

Glioblastoma is the most common and aggressive primary malignant brain tumor with poor prognosis. Novel immunotherapeutic approaches are currently under investigation. Even though magnetic resonance imaging (MRI) is the most important imaging tool for treatment monitoring, response assessment is often hampered by therapy-related tissue changes. As tumor and therapy-associated tissue reactions differ structurally, we hypothesize that biomechanics could be a pertinent imaging proxy for differentiation. Longitudinal MRI and magnetic resonance elastography (MRE) were performed to monitor response to immunotherapy with a toll-like receptor 7/8 agonist in orthotopic syngeneic experimental glioma. Imaging results were correlated to histology and light sheet microscopy data. Here, we identify MRE as a promising non-invasive imaging method for immunotherapy-monitoring by quantifying changes in response-related tumor mechanics. Specifically, we show that a relative softening of treated compared to untreated tumors is linked to the inflammatory processes following therapy-induced re-education of tumor-associated myeloid cells. Mechanistically, combined effects of myeloid influx and inflammation including extracellular matrix degradation following immunotherapy form the basis of treated tumors being softer than untreated glioma. This is a very early indicator of therapy response outperforming established imaging metrics such as tumor volume. The overall anti-tumor inflammatory processes likely have similar effects on human brain tissue biomechanics, making MRE a promising tool for gauging response to immunotherapy in glioma patients early, thereby strongly impacting patient pathway.

## Introduction

Glioblastoma (GBM) is the most common and aggressive primary malignant brain tumor with poor prognosis despite intense standardized therapy. Hence, various novel immunotherapeutic approaches are currently explored to leverage anti-tumor immune responses to improve outcome^1^. However, immunotherapy of GBM is complicated by its highly immunosuppressive tumor microenvironment (TME). The TME is characterized by lower T-cell numbers, high expression of programmed cell death (PD)-1 or its ligands and a high frequency of myeloid cells with an immunosuppressive phenotype compared to other cancer types^2^. As tumor-associated myeloid cells (TAMs) constitute more than 30% of the tumor mass in GBM^3^ and thus are significant contributors to GBM-associated immunosuppression, TAMs represent an attractive therapeutic target. We have recently shown that immunotherapy with the potent toll-like receptor 7/8 (TLR7/8) agonist resiquimod (R848) encapsulated in β-cyclodextrin nanoparticles (CDNP-R848) induces regression of established syngeneic experimental glioma, leading to a sustained anti-tumor response and prolonged survival^4^. Mechanistically, CDNP-R848 treatment activates and re-educates TAMs towards a pro-inflammatory phenotype and achieves tumor regression independent of adaptive immune cells^4^.

Magnetic resonance imaging (MRI) is the most important imaging tool to diagnose brain tumors and to monitor treatment response. It is well appreciated that response assessment in glioma is hampered by therapy-associated phenomena such as pseudoprogression, inflammation or necrosis subsequent to radiation therapy^5^. Even though the current criteria for response assessment in neuro-oncology (RANO) take these into account^6^, differentiation of tumor response from treatment-related tissue changes remains difficult with established, mainly structural MRI techniques.

Here, we hypothesize that immunotherapy-induced inflammatory processes including immune cell influx, edema, increased myeloid-derived matrix metalloproteinase expression, resulting in local ECM degradation, and glioma regression affect the structural organization of the tissue and hence its biomechanics. Biomechanics can be quantified non-invasively via magnetic resonance elastography (MRE)^7^. MRE is sensitive to microstructure and organization of tissue^8^ and renders information on brain tumor composition that are complimentary to established MRI parameters^9–11^. MRE revealed effects of radiation or anti-angiogenic treatment on glioma stiffness in experimental models^12,13^ and further studies hint at a relation between extracellular matrix (ECM) stiffening^14^ or reorganization^15^ and tumor progression. We therefore investigated the effects of myeloid-targeted immunotherapy with CDNP-R848 on biomechanical glioma properties in the syngeneic Gl261 glioma model. We compared biomechanics to the apparent diffusion coefficient (ADC) and fractional anisotropy (FA), which are well characterized metrics derived from MR diffusion tensor imaging (DTI), as both techniques inform about different aspects of the cerebral microstructure, and standard structural MRI. Tumor response was further analyzed by histology and light sheet microscopy (LSM) to establish the cellular and structural ‘ground truth’ of MRE and MRI metrics. Our study shows that MRE is a promising tool for tumor monitoring and response assessment in glioma immunotherapy, with superior performance compared to clinically used MRI techniques.

## Results

### CDNP-R848 treatment has high efficacy and induces tumor regression in 78% of mice

**Figure 1:**
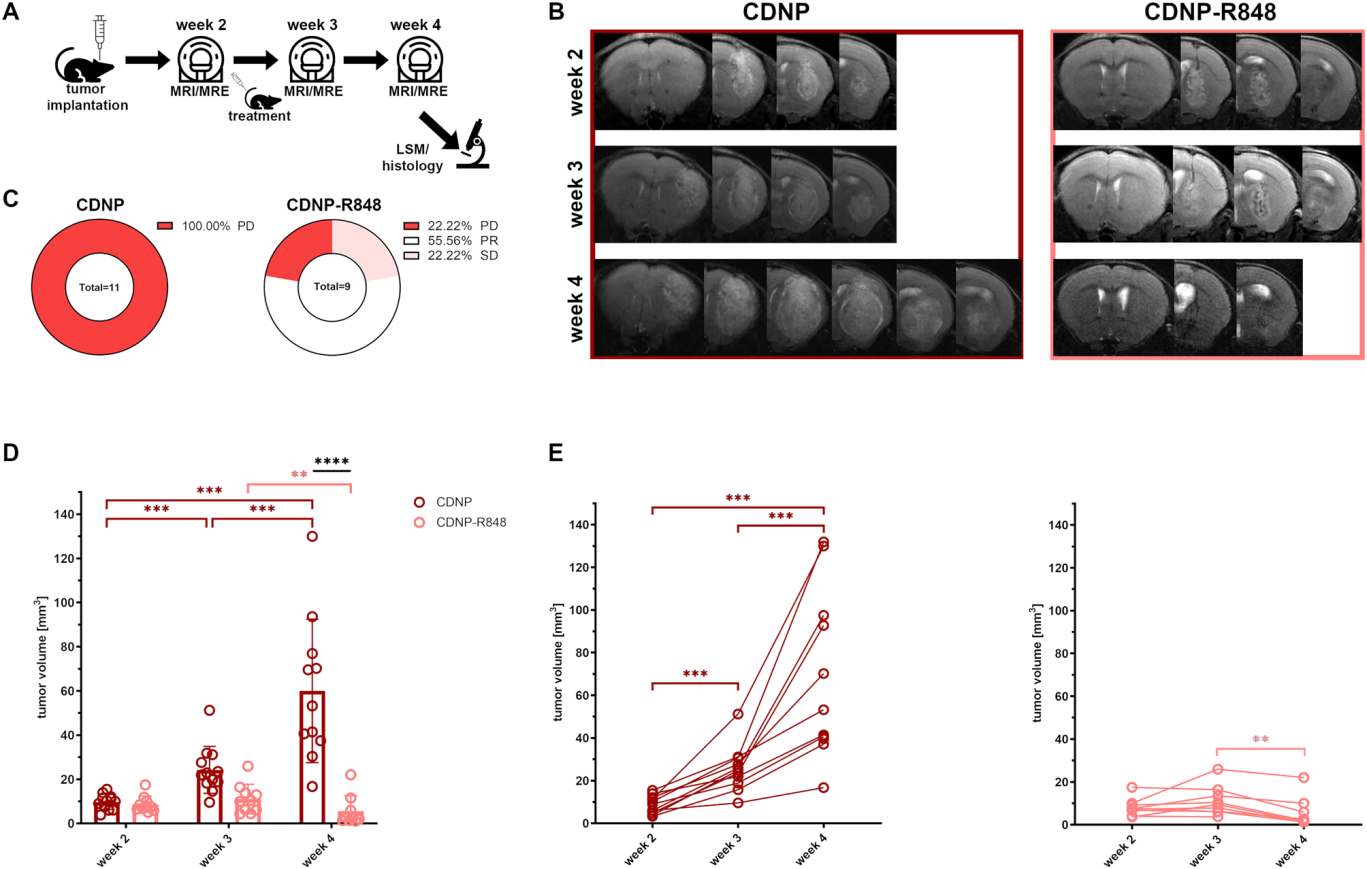
CDNP-R848 treatment leads to tumor regression. The study design is shown in (**A**). Consecutive axial T2w MRI images that show the Gl261 glioma of a CDNP vehicle control (**B**, left) and a CDNP-R848 treated animal (**B**, right) are provided. Of note, MR images are not displayed in radiologic convention to match the LSM and histology data presented in this manuscript (i.e., the right hemisphere is on the right side of the image). Gl261 glioma can be identified as heterogeneously T2w-hyperintense mass in the right striatum. Progressive increase in glioma volume is already visually perceptible in the vehicle control on the left. In contrast, immunotherapy led to tumor regression as visible on the right. This visual perception was confirmed when assessing tumor response according to the iRANO criteria (**C**). Glioma progressed in all CDNP vehicle controls (PD = %Volume_MRIweek4–MRIweek2_ ≥ 40%; **C**, left), while the majority of CDNP-R848 treated mice presented either with partial response (PR = %Volume_MRIweek4–MRIweek2_ ≤ − 65%) or stable disease (SD = %Volume_MRIweek4–MRIweek2_ > − 65% and < 40%; **C**, right). Week-wise comparison of mean tumor volumes (**D**) shows a significant and progressive increase in CDNP vehicle controls (dark red bars, dots and brackets). The temporal evolution of tumor volumes per individual animal are displayed in (**E**). In CDNP-R848 treated animals, tumor volume plateaued when comparing weeks 2 and 3 and then significantly decreased (light red bars, dots and brackets). Tumor volume of both groups only differed significantly in week 4 (black line). Asterisks indicate level of significance based on adjusted p-values derived from a mixed-effects analyses followed by Šídák’s or Tukey’s multiple comparisons test, respectively.

First, we tested if CDNP-R848 treatment can control the growth of established orthotopic gliomas in the syngeneic murine Gl261 glioma model (Fig. 1A). Differences in glioma volume between groups were visually apparent on structural T2w images (Fig. 1B) and further quantified by tumor volumetry. Tumor response to treatment was assessed based on the relative change in tumor volume adapted from the clinically used iRANO criteria^4,16,17^. All animals in the CDNP vehicle control group showed progressive disease (11/11 mice; 100%; PD = %Volume_MRIweek4–MRIweek2_ ≥ 40%), while CDNP-R848 treatment resulted in 56% partial response (5/9 mice; PR = %Volume_MRIweek4–MRIweek2_ ≤ − 65%), 22% stable disease (2/9 mice; SD = %Volume_MRIweek4–MRIweek2_ > − 65% and < 40%) and 22% progressive disease (2/9 mice; Fig. 1C–E). Week-wise comparisons of mean tumor volumes showed a progressive tumor volume increase in animals receiving CDNP vehicle (mean tumor volumes in week 2, 3 and 4: 9.77 mm^3^, 24.27 mm^3^ and 60.02 mm^3^; p < 0.001 for all week-wise comparisons; Fig. 1D). This progressive increase was observable in all animals of this group (Fig. 1E). In contrast, CDNP-R848 immunotherapy first stabilized tumor volume in week 3 and then led to glioma regression in week 4 (tumor volumes in week 2, 3 and 4: 8.74 mm^3^, 11.07 mm^3^, 5.56 mm^3^; p < 0.01 when comparing week 3 and 4; Fig. 1D). Even animals that were classified as non-responders based on the adapted iRANO criteria, which require the evaluation of tumor volume change at the end of therapy in week 4 with respect to baseline in week 2, presented with a decreasing glioma volume from week 3 to week 4 (Fig. 1E). Tumor volume was significantly larger in CDNP vehicle controls than in CDNP-R848 treated animals in week 4 (p < 0.0001; Fig. 1D) but did not differ between groups in weeks 2 and 3.

### The microstructure of Gl261 glioma differs from contralateral normal-appearing brain parenchyma

Next, we compared diffusion parameters and biomechanical metrics as proxies of the Gl261 glioma microstructure to normal appearing brain parenchyma in the contralateral hemispheres of CDNP vehicle controls and animals receiving CDNP-R848 immunotherapy.

**Figure 2:**
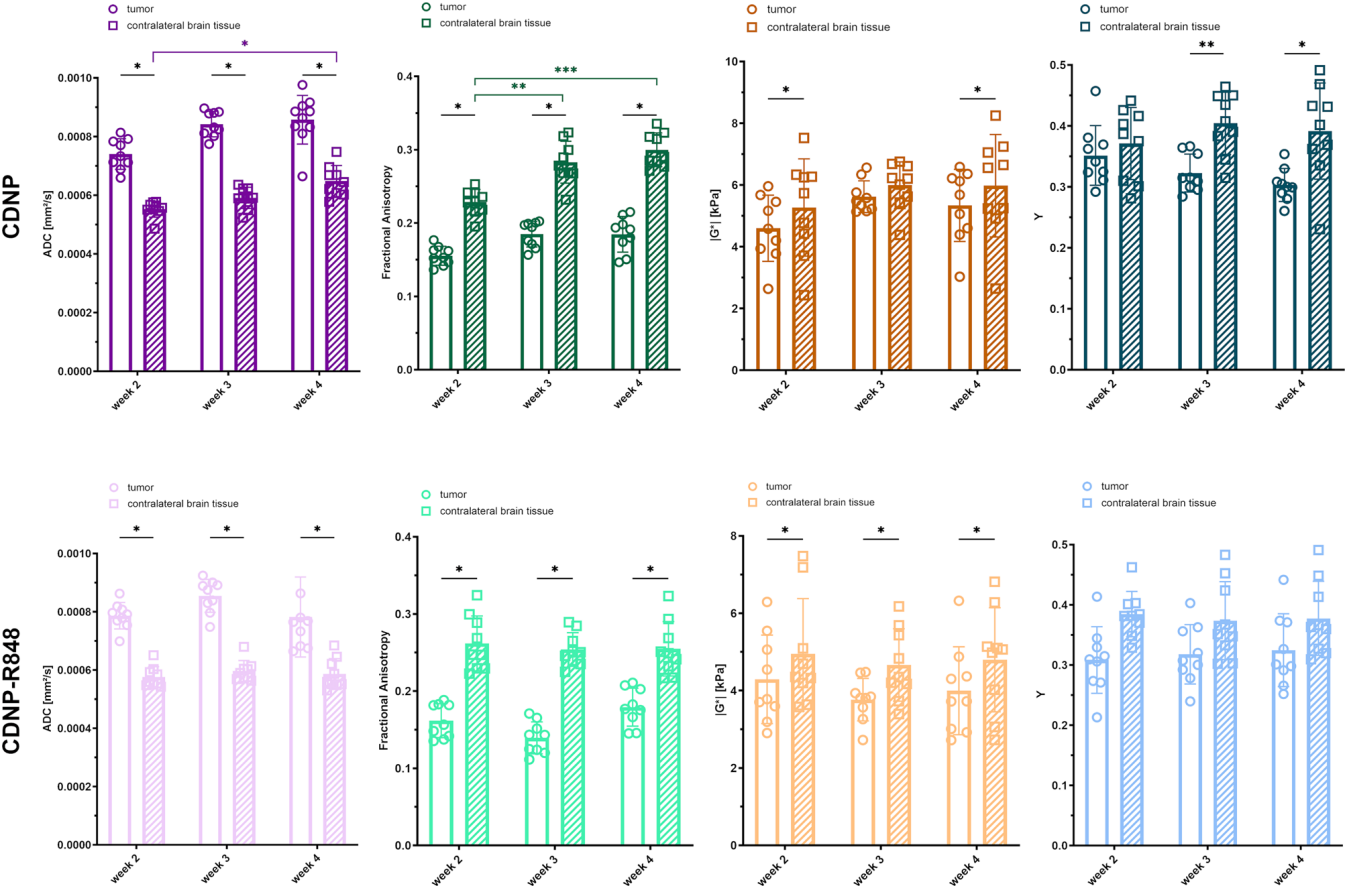
Diffusion and biomechanical properties of Gl261 glioma are different from healthy brain tissue. Mean values of ADC, FA, stiffness |G*| and phase angle Y in Gl261 glioma (dots and unfilled bars) were compared to mean values in the contralateral normal appearing brain tissue (squares and striped bars) of animals receiving the CDNP vehicle (top row, dark colors) or the CDNP-R848 immunotherapy (bottom row, light colors). Asterisks indicate level of significance based on adjusted p-values derived from paired t-tests corrected for multiple testing and from a mixed-effects analysis followed by Tukey’s multiple comparisons test, respectively.

Mean tumor ADC was significantly higher than tissue in the contralateral hemisphere in both groups at all time points (Fig. 2). In CDNP vehicle controls, ADC increased in the contralateral hemisphere when comparing weeks 2 and 4 (mean ADC in the contralateral brain tissue of CDNP vehicle controls in weeks 2, 3 and 4: 0.55 × 10^−3^ mm^2^/s, 0.59 × 10^−3^ mm^2^/s and 0.65 × 10^−3^ mm^2^/s; p < 0.05 when comparing week 2 and week 4; Fig. 2). This increase was not present in CDNP-R848 treated animals (mean ADC in the contralateral brain tissue of CDNP-R848 treated animals in weeks 2, 3 and 4: 0.58 × 10^−3^ mm^2^/s, 0.60 × 10^−3^ mm^2^/s and 0.59 × 10^−3^ mm^2^/s; p > 0.05 when comparing week 2 and week 4; Fig. 2).

Gl261 glioma had a lower FA than contralateral brain tissue in both groups at all time points (Fig. 2). Similar to ADC, FA progressively increased in the contralateral hemisphere of animals receiving the CDNP vehicle (mean FA in the contralateral brain tissue of CDNP vehicle controls in weeks 2, 3 and 4: 0.23, 0.28 and 0.30; p < 0.01 when comparing weeks 2 and 3 and p < 0.001 when comparing weeks 2 and 4; Fig. 2), but did not change over time in CDNP-R848 animals (mean FA in the contralateral brain tissue of CDNP-treated animals in weeks 2, 3 and 4: 0.26, 0.25 and 0.25; p > 0.05 for all comparisons).

Tumors were overall softer than contralateral brain tissue in both groups. Only animals receiving the CDNP vehicle presented with a glioma stiffness equal to the contralateral brain parenchyma in week 3 (mean tumor |G*|: 5.62 kPa vs. mean |G*| of normal appearing brain tissue: 5.99 kPa; p > 0.05; Fig. 2). Stiffness of the contralateral brain tissue did not change over time (mean |G*| in the contralateral hemisphere of CDNP vehicle controls in weeks 2, 3 and 4: 5.27, 5.99 and 5.98 kPa and of CDNP-R848 treated animals in weeks 2, 3 and 4: 4.95, 4.66 and 4.80 kPa; p > 0.05 for all comparisons; Fig. 2).

The mean phase angle Y of glioma in the CDNP vehicle control group was lower than in the contralateral brain parenchyma in weeks 3 and 4 (mean Y of tumor in weeks 3 and 4: 0.32 and 0.30 vs. 0.40 and 0.39 in normal appearing brain tissue; p < 0.01 in week 3 and p < 0.05 in week 4; Fig. 2). This difference was not present in animals receiving CDNP-R848 immunotherapy. The phase angle remained stable in the contralateral brain parenchyma over time (mean Y in the contralateral hemisphere of CDNP vehicle controls in weeks 2, 3 and 4: 0.37, 0.40 and 0.39 and of CDNP-R848 treated animals in weeks 2, 3 and 4: 0.38, 0.37 and 0.38; p > 0.05 for all comparisons; Fig. 2).

### Tumor stiffness differs between groups during the effector and the clearing phase of CDNP-R848 immunotherapy

As tumor volume differentiated treatment groups only one week after completion of the treatment cycle in week 4 and as heterogeneous MRI appearance including pseudoprogression is a known phenomenon in immunotherapy of human glioma^5,18^, we investigated whether tumor response could be predicted using diffusion MRI or MRE.

**Figure 3:**
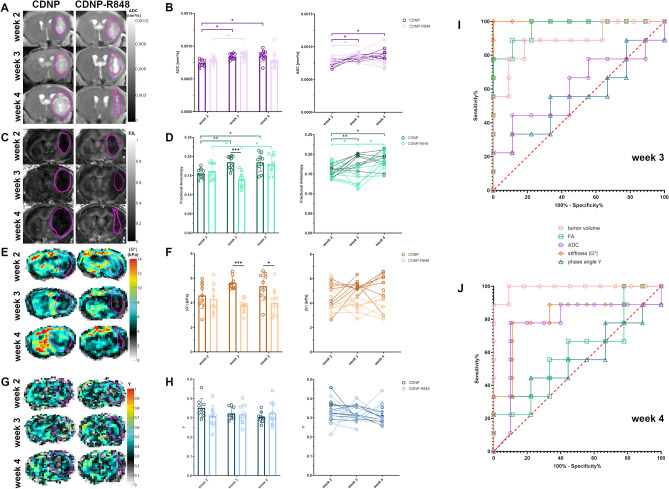
Tumor stiffness early differentiates treated animals and controls and outperforms MRI metrics. (**A**) shows the evolution of ADC in animals receiving the CDNP vehicle and the CDNP-R848 immunotherapy, respectively. ADC progressively increases in CDNP vehicle controls (**B**, dark purple dots, bars and brackets), while in CDNP-R848 treated animals ADC only increased from week 2 to week 3 and stabilized thereafter (**B**, light purple dots, bars and brackets). Mean ADC values did not differ between groups. Exemplary images demonstrating the evolution of FA in the same animals are shown in (**C**). The comparison of mean FA in the glioma of both groups (**D**) revealed an initial increase in FA in CDNP vehicle controls (dark green dots, bars and brackets). CDNP-R848 immunotherapy led to a decrease in FA, which was reversible in week 4 (light green dots, bars and brackets). Mean FA values of both groups differed in week 3 (black line). Elastograms of tumor stiffness |G*| for these animals are shown in (**E**). Mean glioma stiffness did not change over time, but dynamic changes can be appreciated in individual animals (**F**). When comparing both groups, CDNP-R848 treated glioma were softer than tumors in vehicle controls (black lines) in the therapeutic effector phase in week 3 as well as in the clearing phase in week 4. Exemplary elastograms of the phase angle Y are provided in (**G**). Mean tumor phase angle did not change over time and was similar in both groups (**H**). ROC analyses were performed separately for the effector phase (**I**) and the clearing phase (**J**). Gl261 glioma are encircled in pink on all maps. Asterisks indicate level of significance based on adjusted p-values derived from a mixed-effects analyses followed by Šídák's or Tukey’s multiple comparisons test, respectively.

ADC progressively increased in animals receiving the CDNP vehicle only (mean tumor ADC in weeks 2, 3 and 4: 0.74 × 10^−3^ mm^2^/s, 0.84 × 10^−3^ mm^2^/s and 0.86 × 10^−3^ mm^2^/s; p < 0.05 each; Fig. 3A,B), while an initial increase of ADC from week 2 to week 3 was followed by a stabilization in animals receiving CDNP-R848 immunotherapy (mean tumor ADC in weeks 2, 3 and 4: 0.79 × 10^−3^ mm^2^/s, 0.85 × 10^−3^ mm^2^/s and 0.78 × 10^−3^ mm^2^/s; p < 0.05 when comparing week 2 and week 3; Fig. 3A,B). When looking at the temporal evolution of ADC in individual animals (Fig. 3B), ADC tended to decrease in the majority of CDNP-R848 treated glioma from week 3 to week 4. Mean tumor ADC was not significantly different when comparing both groups (Fig. 3B).

In CDNP vehicle controls, mean FA of Gl261 glioma increased in week 3 and then remained stable overall, even though a further slight increase was observable in some animals (mean tumor FA in weeks 2, 3 and 4: 0.16, 0.18 and 0.18; p < 0.01 when comparing weeks 2 and 3 and p < 0.05 when comparing weeks 2 and 4; Fig. 3C,D). In contrast, CDNP-R848 administration caused a decrease of FA in week 3 (mean tumor FA in weeks 2 and 3: 0.16 and 0.14; p < 0.05), which was followed by an increase in all animals (mean tumor FA in week 4: 0.18; p < 0.05; Fig. 3C,D). Mean tumor FA values differed in the therapeutic effector phase in week 3 with CDNP-R848 treated glioma exhibiting lower FA-values compared to vehicle controls (p < 0.001; Fig. 3D).

Interestingly, mean tumor stiffness as quantified by the magnitude of the complex valued shear modulus |G*| was significantly lower in CDNP-R848 treated gliomas than in CDNP vehicle controls in weeks 3 and 4 (mean tumor |G*| in CDNP vehicle controls: 4.60, 5.62 and 5.34 kPa and in CDNP-R848 treated mice: 4.29, 3.77 and 4.00 kPa in week 2, 3 and 4, respectively; Fig. 3E,F). This was most pronounced during the effector phase of the therapy in week 3 (p < 0.001) and persisted in the clearing phase after the end of therapy in week 4 (p < 0.05; Fig. 3F). Glioma stiffness increased from baseline to week 3 in the majority of animals receiving the CDNP vehicle. This increase continued in several animals (Fig. 3F). In contrast, half of the CDNP-R848 treated animals presented with a decreasing tumor stiffness from week 2 to week 3, while the other half with exception of one animal showed this softening from week 3 to week 4 (Fig. 3F).

The analysis of the phase angle Y in the tumors showed no differences between groups or time points (mean tumor Y in CDNP vehicle controls: 0.35, 0.32 and 0.30 and in CDNP-R848 treated mice: 0.31, 0.32 and 0.32 in week 2, 3 and 4, respectively; p > 0.05 each; Fig. 3G,H), indicating that the elastic and viscous contributions to |G*| change in a similar way.

We next tested which parameter differentiated best between glioma of vehicle controls and CDNP-R848 treated animals. ROC analyses were performed for the effector phase (week 3) and the clearing phase (week 4) of CDNP-R848 immunotherapy (Fig. 3I,J). In the effector phase, tumor stiffness discriminated best between controls and treated mice and outperformed FA, tumor volume, ADC and the phase angle Y (Fig. 3I, Table 1). In the clearing phase, tumor volume differentiated treated animals and controls better than tumor stiffness, ADC, FA or phase angle (Fig. 3J, Table 1).

**Table 1:** Criterion values and coordinates of the ROC analysis regarding treatment response.

	AUC	95% CI	Sensitivity (%)	95% CI	Specificity (%)	95% CI	Youden-Index	Criterion
Effector phase (week 3)								
Tumor volume	0.89	0.74–1	88.89	56.5–99.43	81.82	52.3–96.77	0.71	< 17.60 mm^3^
FA	0.96	0.89–1	88.89	56.5–99.43	88.89	56.5–99.43	0.78	< 0.17
* ADC*	0.60	0.33–0.88	66.67	35.42–87.94	55.56	26.67–81.12	0.22	> 0.0008 mm^2^/s
|G*|	1	1–1	100	70.09–100	100	70.09–100	1	< 4.81 kPa
Y	0.56	0.28–0.83	55.56	26.67–81.12	66.67	35.42–87.94	0.22	< 0.31
Clearing phase (week 4)								
Tumor volume	0.99	0.96–1	100	70.09–100	90.91	62.26–99.53	0.91	< 29.57 mm^3^
FA	0.59	0.32–0.86	66.67	35.42–87.94	55.56	26.67–81.12	0.22	< 0.19
ADC	0.77	0.53–1	77.78	45.26–96.05	90	59.58–99.49	0.68	< 0.0007952 mm^2^/s
|G*|	0.83	0.62–1	88.89	56.5–99.43	66.67	35.42–87.94	0.56	< 4.97 kPa
Y	0.57	0.29–0.85	55.56	26.67–81.12	55.56	26.67–81.12	0.11	> 0.30

AUC, area under the curve; CI, confidence interval.

### Gl261 glioma exhibit biomechanical heterogeneity

**Figure 4:**
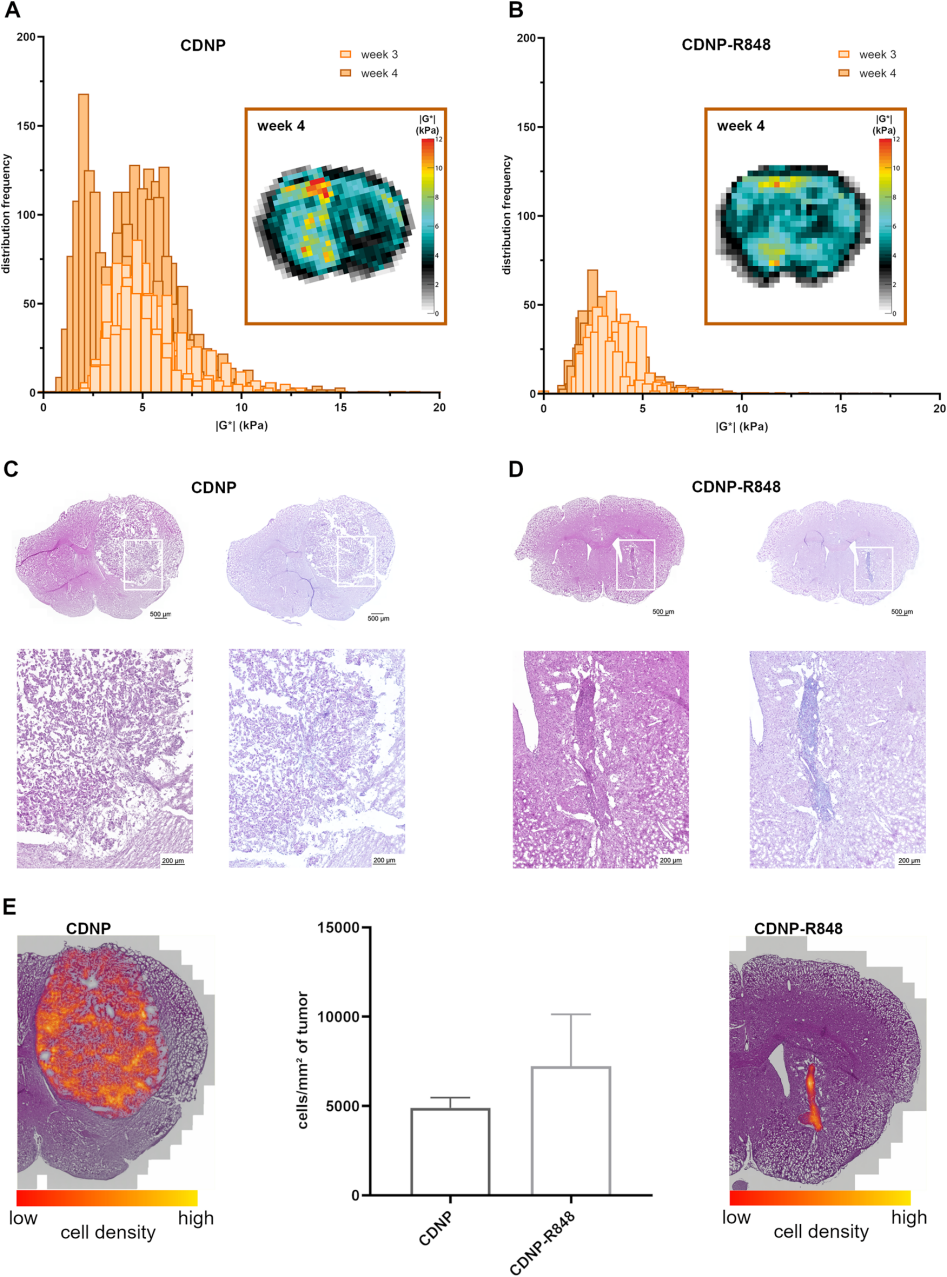
Gl261 glioma exhibit biomechanical heterogeneity reflecting histological composition. Elastograms displaying cerebral stiffness of CDNP vehicle controls and CDNP-R848 treated animals were visually different. This notion is reflected in histograms displaying the frequency distribution of |G*| within the tumors (**A**, **B**). Visual inspection of H&E- and Alcian blue-stained slides (**C**, **D**) revealed that the biomechanical appearance of untreated and treated Gl261 gliomas was reflected in their histological composition. The H&E-stain of animals receiving the CDNP vehicle only showed areas with increased cell density distributed in the tumor area (**C**, left; color-coded cell density map E, left). These regions alternated with patchy accumulation of glycosaminoglycans and mucopolysaccharides stained blue in the Alcian blue-staining (**C**, right). In CDNP-R848 treated gliomas cell density was homogeneously increased in the tumor area (**D**, left; color-coded cell density map E, right). Similarly, glycosaminoglycan and mucopolysaccharide content in the tumor was increased compared to the healthy surrounding tissue and evenly distributed within the entire tumor region (**D**, right). The boxes in the upper rows of (**C**) and (**D**) mark the areas that are magnified in the respective bottom rows. We additionally quantified the cell density in the tumor area on representative H&E-stained slides (**E**). There was a trend toward a higher cell density per mm^2^ of the tumor area in CDNP-R848 treated animals.

Qualitative analysis of the |G*|-elastograms revealed increasing heterogeneity of tumor stiffness in CDNP vehicle controls, displaying alternating softer and stiffer tumor subregions in week 4 (Fig. 4A). In contrast, CDNP-R848 treated glioma appeared rather homogeneous on elastograms. Histogram plots of the distribution frequencies of |G*| within the tumors confirmed a right-skewed distribution of |G*| with a single peak in both controls and treated mice in week 3. In week 4, CDNP vehicle controls developed a double-peaked distribution with maxima at 2 and 5 kPa, respectively, which was not present in CDNP-R848 treated animals (Fig. 4B). To investigate the cause for these differences, we performed H&E- and Alcian blue-staining to assess overall cell density and ECM integrity, respectively. Qualitative and quantitative analyses of these stains reflected the biomechanical heterogeneity (Fig. 4C–E). In animals receiving the CDNP vehicle only, areas with increased cell density alternated with patchy accumulation of glycosaminoglycans and mucopolysaccharides (Fig. 4C,E). The composition of CDNP-R848 treated gliomas was clearly different: cellular density and glycosaminoglycan and mucopolysaccharide content were increased compared to the healthy tissue, but evenly distributed within the entire tumor region (Fig. 4D,E). Cell density in the tumor area tended to be higher in CDNP-R848 treated animals than in animals receiving the CDNP vehicle only (7230 cells/mm^2^ tumor area vs. 4899 cells/mm^2^ tumor area; p > 0.05).

### MRE is sensitive to CDNP-R848 induced anti-tumor inflammation

**Figure 5:**
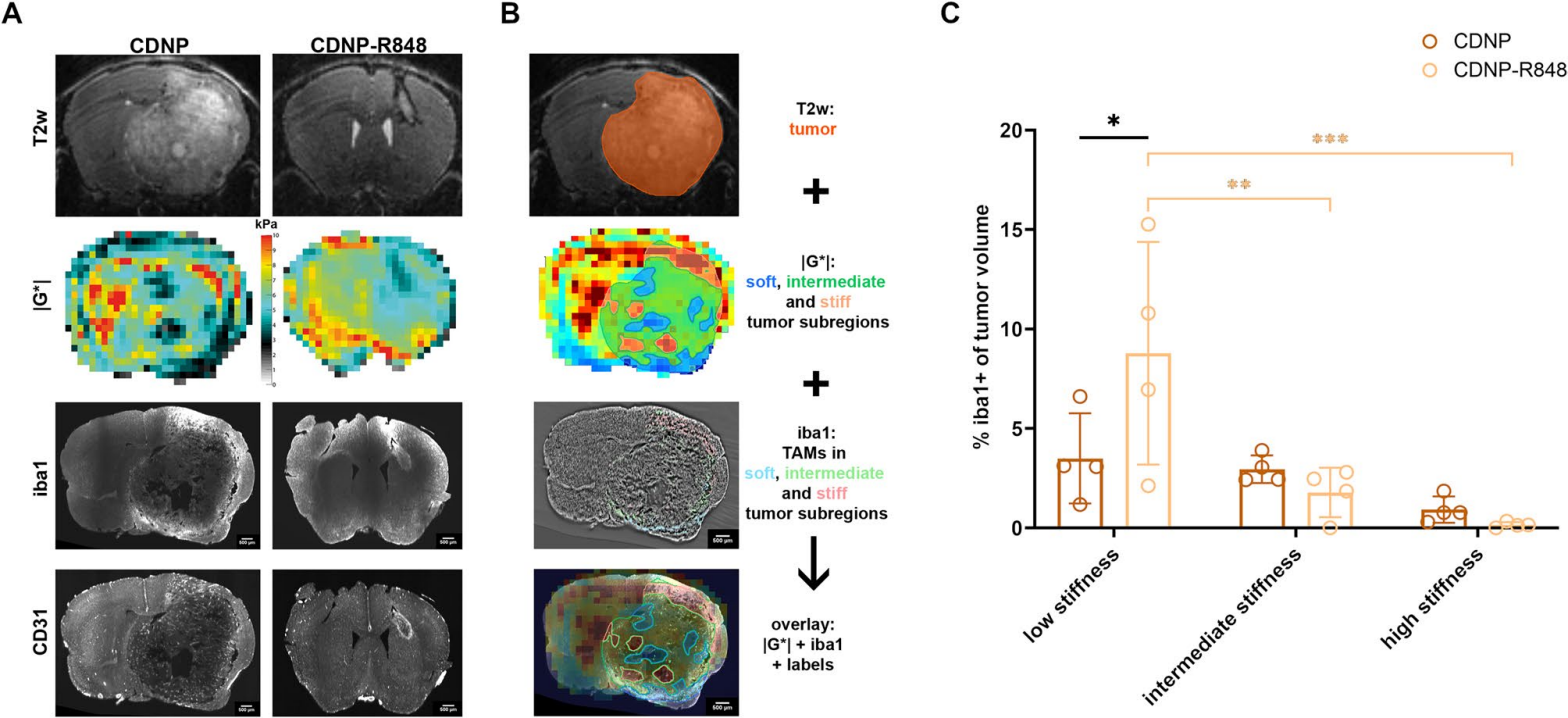
MRE captures effects of CDNP-R848 induced anti-tumor inflammation. To further characterize the mechanism underlying the differences in tumor stiffness between CDNP-R848 treated animals and those that received the CDNP-vehicle only, elastograms and LSM-data were comparatively analyzed (**A**). To determine whether tumor stiffness is influenced by the presence of TAMs and their effects on the tissue, iba1-stained LSM data and |G*|-elastograms were semi-quantitatively compared. For this, |G*|-elastograms and iba1-stained LSM-data were co-registered to the respective T2w 3D images of each animal (**B**). Tumors were segmented on T2w images (B, top row). The tumor label was applied to elastograms, which were used to semi-automatically segment glioma subregions with lower, similar, and higher stiffness than healthy brain parenchyma (**B**, second row). These labels were applied to AHE-filtered iba1-stained LSM data and iba1-positive cells were segmented in glioma subregions (**B**, third row). Finally, the percentage of areas containing iba1-positive cells with regard to the entire tumor area was compared between soft, intermediate and stiff glioma subregions (**B**, bottom row; **C**). Tumor areas with obvious tissue damage, i.e. cracks, holes or missing tissue, were excluded from this analysis. Asterisks indicate level of significance based on adjusted p-values derived from a two-way ANOVA followed by Šídák’s and Tukey’s multiple comparisons test, respectively.

To further characterize the mechanism underlying the differences in tumor stiffness between CDNP-R848 treated animals and those receiving the CDNP-vehicle only, co-registered elastograms and LSM-datasets were first qualitatively assessed. Glioma microvasculature was identified on CD31-stained LSM-datasets. Tumor vessels were larger and more tortuous than normal blood vessels in the contralateral hemisphere (Fig. 5A). In CDNP vehicle controls and in CDNP-R848 treated animals with stable or progressive disease, tumor vessels centripetally penetrated Gl261 gliomas. CDNP-R848 treated animals with tumor response showed pathological vasculature around the borders and to a lesser extent centripetally distributed in the Gl261 residuum. The distribution of TAMs, which were identified on iba1-staining, visually differed between the treatment groups: while animals that received CDNP showed patchy clusters of TAMs at the tumor borders and partially within the tumor core, a homogeneous distribution of TAMs within and in the immediate vicinity of the glioma was observable in mice treated with CDNP-R848 (Fig. 5A). Based on this qualitative assessment, the tumor microvasculature was ruled out as cause of the biomechanical differences between the groups. The homogeneous distribution of pathological blood vessels did not match the heterogeneous stiffness distribution observed in CDNP vehicle controls. To determine whether tumor stiffness is influenced by the presence of TAMs and their effects on the tissue, we semi-quantitatively compared iba1-stained LSM data and |G*|-elastograms. For this purpose, the percentage of tumor regions containing TAMs was compared between glioma subregions exhibiting lower, similar and higher stiffness than healthy brain parenchyma (Fig. 5B). In CDNP vehicle controls, there were no differences in the amount of TAMs between stiff, intermediate and soft Gl261-subregions (p > 0.05 for each comparison; Fig. 5C). In CDNP-R848 treated mice, TAMs were more abundant in tumor areas with low stiffness compared to areas of intermediate and high stiffness (mean percentage of iba1-positive tumor volume in areas with low, intermediate and high stiffness: 8.78, 1.78 and 0.15%; p < 0.01 and p < 0.001 when comparing subregions of low to subregions of intermediate and high stiffness, respectively; Fig. 5C). Moreover, the percentage of TAMs in soft tumor subregions was higher in CDNP-R848 treated animals than in vehicle controls (p < 0.05; Fig. 5C).

## Discussion

Therapy monitoring of glioma is a challenge in neuroradiology and hampered by treatment-associated tissue changes that resemble tumor progression on conventional structural MR images^5^. Even though such limitations are addressed in the current RANO criteria^6^ and quantitative MRI techniques are used along structural imaging^19,20^, definite differentiation between tumor and treatment-related phenomena requires biopsy and histological evaluation or longitudinal follow-up over months. Biopsy is invasive and long follow-up not only carries diagnostic uncertainty but could also delay accurate treatment of recurrent tumors. Thus, there is a high demand for better non-invasive options to support neuroradiological monitoring and clinical decision making for patients with glioma. Here, we show that MRE has the potential to overcome this diagnostic dilemma.

**Figure 6:**
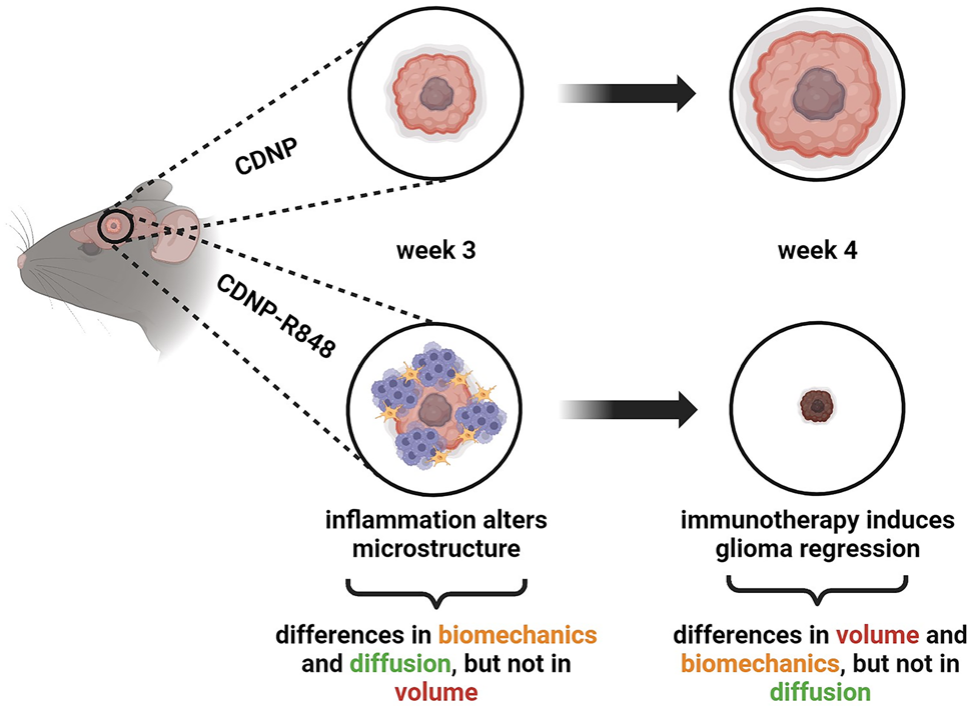
Graphical summary of findings. Immunotherapy with CDNP-R848 re-educates myeloid cells towards a pro-inflammatory phenotype. The following inflammatory processes globally affect the tumor microstructure, which leads to significant differences in diffusion properties (FA) and in biomechanics between untreated and treated gliomas well before changes in tumor volume are evident. Ultimately, this specific treatment causes regression of GL261-glioma. This is reflected in significantly smaller and softer tumors in treated animals, while diffusion properties did not differ between treated and untreated glioma after completion of therapy. This figure was created with www.biorender.com.

We have recently shown the efficacy of the TLR7/8 agonist resiquimod when encapsulated in CDNP in a preclinical glioma model^4,21^. Mechanistically, CDNP-R848 causes a profound shift towards a pro-inflammatory macrophage phenotype that is accompanied by changes in the chemokine and cytokine secretion as well as increased production of reactive oxygen species, ultimately leading to a considerable re-shaping of the TME^4^. Here we showed that this specific treatment globally affects the tumor microstructure. It led to significant differences in diffusion properties (FA) and in biomechanics (|G*|) between untreated and treated gliomas, well before changes in tumor volume became evident. Figure 6 provides a graphical summary of this concept.

Diffusion MRI has been used to characterize brain tumors^22^ and is implemented in routine clinical protocols^23^. ADC is inversely associated with cell density and several studies have demonstrated lower ADC in glioma compared with surrounding brain tissue or therapy-associated tissue changes^5^. In our study, ADC of Gl261 glioma is higher than in the contralateral brain tissue despite histologically proven increased cell density. In this regard, it is important to note, that our diffusion data were acquired at an experimental scanner with very high gradient strength compared to clinical MRI scanners. Thus, ADC in our experiments predominantly reflects intracellular diffusion processes in contrast to mainly extracellular diffusion captured in a clinical setting^24^. The high tumoral ADC may thus be explained by the larger size of glioma cells compared to other cell types in the brain. Despite the sufficient sensitivity of diffusion MRI to detect high glioma cellularity, it has inadequate specificity to clearly differentiate tumor progression and therapy-associated tissue changes due to high intratumoral heterogeneity and confounding factors such as hemorrhages or inflammation^5^. Therefore, the evaluation of ADC is not included in standard glioma response assessment^6^. Our findings are in line with this clinical experience, as ADC was not different between treated and untreated glioma and did not capture early therapy-mediated anti-tumor inflammation. DTI is an established method to assess tissue microstructure with FA being generally attributed to the organization of white matter tracts. Disturbance or disruption of this highly organized structure leads to a decrease in FA, which is well perceivable in the comparison of mean FA in Gl261 glioma to tissue in the contralateral hemisphere. Moreover, this has been demonstrated in a preclinical model of hydrocephalus, for example, where transependymal edema was accompanied by low FA^25^. A clinical study in patients with brain metastases found that lower FA in the peritumoral region correlated with increased T-cell densities and independently predicted longer overall survival^26^. Our results are in line with these findings, as FA was significantly lower in CDNP-R848 treated animals during the effector phase of the therapy in week 3. After completion of therapy, i.e., during the clearing phase, FA values of treated tumors returned to values similar to untreated glioma. The transient decrease of FA can be explained by combined effects of inflammatory cell accumulation and edema caused by CDNP-R848.

However, biomechanics seem to also be sensitive to the effects of immunotherapy on Gl261 glioma as tumor stiffness discriminated between animals receiving CDNP-R848 and CDNP vehicle during both the therapeutic effector phase and the clearing phase. The high sensitivity of biomechanical features to brain inflammation in general has been shown in patients with multiple sclerosis (MS) and its animal model, experimental autoimmune encephalomyelitis (EAE). Global brain stiffness of patients with MS is lower than in age-matched healthy controls^27–29^ and even very early inflammatory activity led to a decrease in mean cortical viscoelasticity in mice with EAE^30^. In this study, we were able to relate glioma softening to the presence of iba1-positive TAMs. The percentage of TAMs in soft tumor subregions of CDNP-R848 treated animals was significantly higher than in subregions of intermediate and high stiffness, and also significantly higher than in soft subregions of untreated glioma. Softening, as measured with MRE, seems to be a direct indicator of anti-tumor inflammation including accumulation of immune cells, edema, ECM reorganization and glioma cell death. Thus, glioma biomechanics could serve as an imaging proxy to assess response to immunotherapy.

Our findings are consistent with a previous study showing that the magnitude of cerebral elasticity reduction in EAE-animals correlated with F4/80 gene expression as a macrophage/microglia marker in neuroinflammation^31^. In another EAE-study, a similar relation between softening and local immune cell infiltration has been demonstrated^32^ supporting our hypothesis that not only the presence of TAMs, but the inflammatory processes following CDNP-R848 induced re-education of TAMs are detectable by changes in biomechanics.

In line with other murine glioma models^12,33,34^ and various human brain tumors^10^, Gl261 glioma are overall softer than contralateral normal appearing brain tissue. The lacking difference in stiffness observable in CDNP vehicle controls in week 3 can be explained by a transient tumor stiffening. This stiffening might be attributable to densely packed viable Gl261 cells within the tumor bulk similar to previous work, in which areas with high glioma cell density were stiffer than necrotic or hemorrhagic tumor subregions in a xenograft model of GBM^34^. The lower phase angle in Gl261 glioma compared to contralateral brain tissue of CDNP vehicle controls in weeks 3 and 4 indicates a shift towards more elastic than viscous contributions to tumor viscoelasticity. A decreasing phase angle hinted at an increased invasiveness during anti-angiogenic treatment of murine xenograft GBM^13^. It is conceivable, that the decreased phase angle of Gl261 glioma compared to the contralateral hemisphere in CDNP vehicle controls, which was not present in animals receiving CDNP-R848 immunotherapy, is caused by a more aggressive growth of untreated tumors. MRE also revealed biomechanical heterogeneity—not identifiable in conventional T2w-images—of developed gliomas in animals receiving the CDNP vehicle only. This heterogeneity was reflected in the histological composition of tumors as areas with high cell density alternated with patchy accumulation of extracellular matrix components as well as with tissue damage presumably caused by necrosis and hemorrhage. The ability to delineate histologically distinct glioma subregions has already been demonstrated in a patient-derived xenograft mouse model of GBM^34^ and in patients with glioma^15^. Here, stiff tumor subregions were associated with ECM reorganization and expression patterns of ECM proteins in such stiff tumor areas correlated with a shorter overall survival of patients^15^. Hence, MRE can complement established MRI techniques by providing additional information on the structural tumor organization, which are otherwise not accessible non-invasively.

Progressive changes of both ADC and FA were observable in the normal-appearing brain tissue of CDNP vehicle controls. This seems to be non-tumor specific as Gl261-glioma grow bulky and do not infiltrate the surrounding brain tissue or the contralateral hemisphere^35^. Potentially, the observed changes can be ascribed to effects of tissue displacement by large tumors or extension of tumor-growth related white matter tract damage to the contralateral hemisphere. MRE-parameters in the contralateral hemisphere did not change over time and were not affected by non-tumor specific tissue changes.

Limitations of our study include that the MRE acquisition did not have full brain coverage but was limited to 9 slices due to time constraints of our multiparametric imaging protocol. While smaller tumors were entirely covered, the outer parts of large tumors in CDNP vehicle controls in week 4 were not recorded. We used our recently established fiducial-based approach to co-register MRI, MRE and LSM-data^35^ and insufficient registration could potentially bias our results, but this affects both controls and treated animals to a similar extent. Additionally, as the study was designed longitudinally, we could not compare imaging and LSM- or histological data at all time points. Our design did not allow to investigate potential differences in response dynamics to CDNP-R848 immunotherapy. A decreasing tumor volume from week 3 to 4 in animals classified as CDNP-R848 non-responders and glioma softening occurring from week 2 to 3 in some animals and in week 3 to 4 in the remainder suggest a potential “delayed” response, which we cannot further corroborate with our data. These aspects should be investigated further with a cross-sectional design and a prolonged time of observation after the end of therapy, respectively. Furthermore, models with known resistance to an immunotherapeutic regime should be investigated to observe the expected absence of softening. Moreover, our group sizes were too small to perform meaningful analyses of subgroups. In that regard, further studies allowing for a comparison of imaging metrics, percentage of TAMs and histological markers in CDNP-R848 responders and non-responders would be important to investigate the value of biomechanics for treatment monitoring. Finally, clinical studies are needed to investigate if our findings are translatable to glioma patients treated with immunotherapies^36,37^.

In conclusion, microstructural imaging parameters such as FA and tumor stiffness are sensitive and complementary markers for immunotherapy-induced inflammation leading to glioma regression. These parameters indicated therapy response during the therapeutic effector phase well before structural tumor regression had occurred, thereby outperforming conventional MRI metrics such as tumor volume and ADC. Moreover, biomechanical heterogeneity reflected the histological tumor composition and hence MRE can help to non-invasively characterize glioma subregions. Tumor growth and the therapy-induced anti-tumor inflammatory processes in mice are likely to have similar effects on human brain tissue biomechanics. Thus, MRE in addition to DTI is a promising novel tool for the assessment of glioma response to immunotherapy. Non-invasive imaging proxies for the quantification of early response or resistance to immunotherapy are currently not available in humans pushing clinical decision making to several months after therapy initiation. Microstructural tumor assessment with DTI and biomechanics carries the potential to overcome this current diagnostic dilemma by impacting patient pathway much earlier.

## Methods

### Animal model

All experiments were performed according to the institutional animal research guidelines and the Society for Laboratory Animal Science (GV-SOLAS) guidelines and were approved by the regional administrative authority (Regierungspräsidium Karlsruhe, Germany; permit numbers: G-27/17 and G-35/22). Experimental procedures and results are reported in accordance with the ARRIVE guidelines. Gl261 cells were purchased from the National Cancer Institute and cultured in Dulbecco’s modified Eagle’s medium (DMEM) supplemented with 10% fetal bovine serum (FBS), 100 U/ml penicillin and 100 µg/ml streptomycin (all from Sigma-Aldrich, Taufkirchen, Germany) at 37 °C, 5% CO_2_. The cell line was routinely tested by the multiplex cell contamination test^38^. Specific and opportunistic pathogen free (SOPF) female 6–10 weeks old C57BL/6 J mice (Janvier Labs, Le Genest-Saint-Isle, France) were used for the experiment. Animals were housed with 12 h/12 h-light/dark-circle and food and water ad libitum. After one week of acclimatization, 1 × 10^5^ Gl261 cells in 2 µl phosphate buffered saline (PBS, Sigma-Aldrich) were stereotactically implanted in the right striatum (2 mm right lateral of the bregma, 1 mm anterior of the coronal suture, injection depth: 3 mm) using a Hamilton syringe with a fine step motor as previously described^4,17^. Mice were anesthetized with ketamine/xylazine during the procedure.

### Study design

A total of 20 mice were randomized into a therapy group and a control group based on tumor volumes assessed on standard T2w MRI. CDNP-R848 was formulated as previously described^21^ and used as immunotherapeutic treatment. The therapy group received three doses of 100 µl CDNP-R848 i.v. on day 14, 17 and 20 after tumor implantation. The other group received 100 µl CDNP i.v. on the same days as vehicle control. MRI and MRE imaging were conducted 12, 19 and 26 days after tumor implantation, i.e. at baseline pre-treatment (week 2), during the therapeutic effector (week 3) and in the clearing phase after completion of treatment (week 4). Thereafter, animals were anesthetized with a lethal dose of ketamine/xylazine and transcardially perfused with PBS followed by 4% paraformaldehyde (PFA). Then, brains of a subgroup of eight animals were harvested and prepared for whole brain clearing, LSM and histology after rehydration. MRI and MRE data were used for response assessment and for the analysis of tumor volume, FA and MRE parameters. Analyses of LSM and histology data were performed separately. Figure 1A provides an overview of the study design.

### MR-imaging

Multiparametric MRI and MRE were performed on a 9.4 T Bruker small animal MRI scanner (BioSpec 94/20 USR, Bruker BioSpin GmbH, Ettlingen, Germany; gradient strength 675 mT/m) using an 8.4 cm body coil for transmission and a 2 × 2 surface array coil for reception. Anesthesia was induced with up to 4% isoflurane (Baxter, Unterschleißheim, Germany) in 100% O_2_ and maintained with 1–1.5% isoflurane in 100% O_2_. Respiration rate was constantly monitored. Animals were placed prone on either a standard Bruker MRI bed or a custom-build MRE bed, both equipped with a water heating system to maintain body temperature. After standard shimming (global shim and local shim on the brain volume), piloting and acquisition of a B0 field map, an axial 2D T2w rapid acquisition with relaxation enhancement (RARE), an isotropic 3D T2w RARE with a spatial resolution of 120 µm and an axial echo-planar imaging (EPI) DTI sequence with a b-value of 1500 s/mm^2^, 30 directions, a gradient duration of 3 ms and a gradient separation of 9 ms were acquired. 3D multi-slice multi-echo (MSME) MRE at an isotropic spatial resolution of 300 µm was performed as previously described^13,39^. Briefly, the head of the animal was fixated on a movable head cradle. The head cradle was coupled to an external electromagnetic shaker (Mini-shaker 4810, Brüel & Kjaer, Naerum, Denmark) through a flexible nylon rod. The shaker generated vibrations at a frequency of 900 Hz, which were transmitted via the rod to the head cradle eliciting mechanical shear waves in the animal’s brain. Vibrations were synchronized to the MRE sequence by capturing a TTL trigger pulse from the MRI scanner with a function generator that controlled the shaker. A spin-echo MRE sequence with an echo time (TE) of 27 ms was chosen to ensure sufficient phase accrual and to provide sufficient time for the motion-encoding gradients. 18 motion-encoding gradient cycles were employed. The multi-slice acquisition was used to prolong the repetition time (TR) and gain signal. Multiple echoes were not utilized. Sequence planning and data acquisition were performed using ParaVision 6 software (Bruker BioSpin GmbH). Detailed sequence parameters can be found in Table 2.

**Table 2:** Parameters of MRI and MRE sequences.

Sequence	TR (ms)	TE (ms)	Flip angle	Averages	Acquisition matrix	FOV (mm)	Slice thickness (mm)	Number of slices	Duration
3D T2w TurboRARE	1800	72.5	90	1	200 × 200	20 × 10 × 12	0.1	120	10 min 48 s
2D T2w TurboRARE; axial	1250	33	90	2	128 × 128	19.2 × 19.2	0.3	9	5 min 20 s
DTI EPI 30 directions; axial	3400	20	90	1	96 × 128	12 × 15	0.7	17	7 min 56 s
MRE MSME; 900 Hz vibration frequency; axial	1500	26.67	90	1	64 × 64	19.2 × 19.2	0.3	9	19 min 12 s

DTI, diffusion tensor imaging; EPI, echo planar imaging; FOV, field of view; MRE, magnetic resonance elastography; MSME, multi slice multi echo; RARE, rapid acquisition with relaxation enhancement; TE, echo time; TR, repetition time.

### Tissue clearing and LSM

Following the last imaging time point, eight animals received an i.v. injection of 10 µg CD31-AF647 (catalog # 102516, BioLegend, Koblenz, Germany) in PBS (total injection volume of 100 µl) as an intravital dye to stain the vasculature. After intracardial perfusion, brains were harvested and optically cleared following the iDISCO protocol^40^, including an anti-ionized calcium binding adaptor molecule 1 (iba1)-staining for macrophages and microglia.

To be specific, the mouse brain samples were dehydrated in a gradient of 40%, 60%, 80% and 100% methanol (catalog # 4627; Carl Roth, Karlsruhe, Germany) solutions in distilled water with 1 h for each step with gentle shaking. After an additional 100% methanol incubation for 1 h, the samples were incubated in a mixed solution with 33% methanol and 67% dichloromethane (vol/vol; catalog # KK47, Carl Roth) overnight at room temperature with gentle shaking. An overnight bleaching step was conducted after 2 times of short wash with methanol by applying freshly prepared 5% H2O2 (catalog # LC-4458, Labochem, Sant’Agata li Battiati, Italy) solution in methanol (vol/vol) at 4 °C. The samples were rehydrated with reversed incubation in 80%, 60%, 40% methanol solutions and in PBS for 2 times, with gentle shaking for 1 h each. After incubation in PBSTx solution containing 0.2% TritonX-100 (Sigma, X100) for 2 times for 1 h each, the samples were incubated in permeabilization solution containing 20% DMSO (vol/vol; catalog # A994, Carl Roth) and 2.3% glycine (wt/vol; catalog # LC-4522, Labochem) prepared in 0.2% PBSTx solution for 3 days at 37 °C with gentle shaking. After a blocking step with 10% DMSO (vol/vol) and 6% goat serum (vol/vol; catalog # 16210072, Gibco/Thermo Fisher Scientific) in 0.2% PBSTx solution for 3 days at 37 °C, the samples were treated with iba1 primary antibody (catalog # 019-19741, Wako, Darmstadt, Germany; 1:250 dilution) in a labeling solution prepared with 5% DMSO (vol/vol) and 3% goat serum (vol/vol) in PBSTw solution containing 0.2% Tween20 (catalog # P2287, Sigma, Darmstadt, Germany) for 8 days at 37 °C with gentle shaking. After washing with 0.2% PBSTw solution for 4 times including one overnight incubation, the samples were treated with goat anti-rabbit-IgG secondary antibody conjugated with Alexa 568 dye (catalog # A11011; Invitrogen/Thermo Fisher Scietific; 1:250 dilution) in 0.2% PBSTw solution added with 3% goat serum for 8 days at 37 °C with gentle shaking. After washing with 0.2% PBSTw solution for 4 times including one overnight incubation, the samples were dehydrated with 50%, 70%, 90%,100% for 1 h each and 100% overnight incubation of methanol solutions. Samples were incubated for 3 h in 33% methanol/67% dichloromethane solution and for 30 min in 100% dichloromethane. Finally, a refractive index matching solution composed of 33% benzyl alcohol (catalog # 24122, Sigma) and 67% benzyl benzoate (vol/vol; catalog # W213802, Sigma) was applied until the samples reached optical transparency. All tissue clearing steps were conducted at room temperature with gentle shaking under a fume hood. The samples were covered with aluminum foil from secondary antibody incubation to prevent fluorescence loss.

Cleared brains were scanned with a light sheet microscope (LCS SPIM, Luxendo-Bruker; Heidelberg, Germany) using a 4.0 × objective lens (Olympus XLFLUOR 4x/340, 0.28 NA) and combined lasers (excitation wavelength at 488, 561, 642 and 685 nm with respective filters and input power of 40 mW each). An effective magnification of 4.4 × with pixel size of 1.46 × 1.46 µm was used for image acquisition and tiling z-stack scans with 6 µm step size were performed to cover the entire brain sample (total acquisition time approx. 60–70 min per brain with exposure times of 100 ms per slice). Image Processor was used for stitching and images were exported as series of tagged image file (TIF) for further analysis. To facilitate correlative MRI-LSM-analysis and to speed up the processing pipeline, LSM-data were downsampled using the bin transform of open-source FIJI software (FIJI/ImageJ for Windows, version 2.0, www.imagej.net^41^) applying a shrink factor of 5 × 5 × 5 (bin method: average), which averages voxels within the 5 × 5 × 5 region into one voxel leading to a downsampled resolution of 7.3 × 7.3 × 30 µm.

### Histology and immunohistochemistry

After LSM data acquisition brains were rehydrated with reversed incubation in 100%, 80%, 60% and 40% methanol (catalog # 4627; Carl Roth) and in PBS with gentle shaking for 1 h. Then, samples were fixed in 4.5% PFA overnight at 4 °C and washed twice with PBS for 30 min each afterwards. Rehydrated brains were placed in 15% and 30% sucrose solution until sample sank to ground, respectively, embedded in TissueTek (catalog #SA62550-01, Science Services, Munich, Germany) and frozen on dry ice. Brains were finally cryo-sectioned with a slice thickness of 7 µm.

Standard H&E- and Alcian blue-staining were performed on frozen tissue sections. For H&E, slides were washed with distilled water, stained with hematoxylin (catalog #9194, Carl Roth) for 6 min, rinsed in tap and in distilled water and counterstained with eosin for 3 min (catalog #9194, Carl Roth). After that, slides were immersed in 70%, 96% and 100% ethanol for 2 min each followed by xylene and mounted. For Alcian blue, a staining kit was used (catalog #1326570001, Sigma). After rinsing with distilled water, slides were stained with Alcian blue solution (pH 2.5) for 15 min and rinsed in running tap followed by distilled water. Slides were placed in 0.1% nuclear fast red-aluminum sulfate solution for 10 min and immersed in an ascending alcohol series (70%, 96% and 100% ethanol, 2 min each), which was followed by xylene and mounted. Images were obtained on Zeiss Axio Scan.Z1 with a 20× magnification.

### Data reconstruction and image analysis

MRE data reconstruction was realized according to published algorithms^7,42^ using dedicated in-house software (ROOT environment, CERN; Meyrin, Switzerland). Briefly, the phase data are unwrapped per slice and aligned between slices. A 4-point Fourier transform was calculated as four wave phases have been acquired and the first harmonic amplitude is used to represent the displacement amplitude. Then, any compression waves are removed using the curl of the displacement data. Spatial derivates are calculated and used to inverse the Helmholtz equation. Color-coded maps of the MRE-parameters (elastograms) were calculated and used for further analyses. The absolute value of the complex shear modulus |G*| (‘viscoelasticity’; referred to as ‘stiffness’) and the phase angle Y were investigated. |G*| comprises measures of both elasticity (shear modulus, G_d_) and viscosity (loss modulus, G_l_) and is calculated as $\left|G*\right|= \sqrt{{{G}_{d}}^{2}+{{G}_{l}}^{2}}$. The relative contribution of elasticity and viscosity to the shear modulus is expressed in the phase angle by $Y= \frac{2}{\pi } \text{atan}(\frac{{G}_{l}}{{G}_{d}})$. At the extremes, a given material is purely elastic (Y = 0) or purely viscous (Y = 1). T2w images were automatically co-registered and displayed as anatomical reference. Elastograms were exported in NIfTI format.

ADC was calculated using a customized script in MATLAB (MATLAB R2020a, 64-bit version for Windows, The MathWorks Inc., Natick, MA, USA) and ADC-maps were exported in DICOM format. FA maps were calculated from DTI raw data with ParaVision 6 software (Bruker BioSpin GmbH) and exported in DICOM format.

Processing and combined analysis of all MRI, MRE and LSM data was performed with open source 3D slicer software (3D Slicer for Windows, versions 4.11.0 and 4.13.0, www.slicer.org^43^). First, all data were brought to NIfTI format. Then, ADC- and FA-maps and elastograms were co-registered to the 3D T2w image of each animal per time point applying a linear rigid transform. Downsampled LSM-data of each animal were co-registered to the respective 3D T2w acquired in week 4 using a manual anatomical-landmark based registration as previously described^35^. Briefly, fiducials were placed using anatomical landmarks clearly identifiable on each dataset, e.g., ventricles, white matter tracts or blood vessels. A warping transform was created and applied. Manual tumor segmentation was performed on T2w images and semi-automatic segmentation of iba1-positive myeloid cell-containing regions on LSM-images was achieved threshold-based. Tumor volume was obtained and mean values of ADC, FA, |G*| and Y were extracted in the tumor as well as in LSM-defined regions of interest. In addition, an area of equal volume as the glioma was segmented in the contralateral hemisphere of each animal on T2w images. Mean values of the diffusion and the MRE parameters in these normal-appearing brain regions were calculated and compared to the respective tumors. The results obtained from tumor volumetry have partly been published previously^4^. All other results concerning DTI-, MRE-, LSM- and histological data have not been published elsewhere.

Cell density of representative H&E-stained slides obtained in the tumor core of animals in both groups was realized using open-source QuPath software (QuPath-0.5.1. for Windows, https://qupath.github.io^44^). A region of interest covering the tumor area was manually defined. The cell detection/density distribution tool was used to obtain cell densities per mm^2^ within the tumor area. Color-coded cell density maps were created with a density radius of 50–100.

To compare the distribution of iba1-positive cells within the tumor to its stiffness, LSM-images were first filtered using the adaptive histogram equalization (AHE^45^) filter to enhance contrast (filter parameters: radius 5 × 5 × 5, alpha = 0.3, beta = 0.3). The generated warping transform was applied to downsampled, AHE-filtered images and transformed data were used for further analysis. As both LSM-data and elastograms were co-registered to T2w 3D data, they could be directly compared. First, areas with iba1-positive cells were semi-automatically segmented threshold-based. Then, elastograms of |G*| were also semi-automatically threshold-based segmented to determine regions with stiffness lower, equal to and higher than healthy brain tissue (“low”: < 4.5 kPa, “intermediate”: 4.5–6.5 kPa and “high”: > 6.5 kPa stiffness). These segmentations were cropped to the T2w-determined tumor area. Areas that were damaged during tissue clearing and appeared as holes or cracks within and around the tumor were semi-automatically threshold-based segmented and excluded from further calculations. Finally, the percentage of iba1-positive volume with respect to the total tumor volume was calculated for each stiffness category.

### Statistical analysis

Statistical analysis was performed with GraphPad Prism (version 9.5.0 for Windows, GraphPad Software, La Jolla, CA, USA). Mean values of diffusion and MRE-parameters in the tumors and in the contralateral brain tissue were compared with paired t-tests corrected for multiple testing. A mixed-effects analysis followed by Šídák's multiple comparisons test was conducted to compare mean values of tumor volume, ADC, FA, |G*| and Y between groups per time point. To evaluate the temporal evolution of these parameters within the groups, a mixed-effects analysis followed by Tukey’s test for multiple comparisons was applied. Tumor volume, ADC, FA, |G*| and Y were further examined by receiver operating characteristics (ROC) analysis regarding tumor response applying the Wilson/Brown method. Sensitivity and specificity were calculated together with the two-sided 95% confidence intervals (CI) for the cutoff-values and the Youden-index. Heterogeneity of |G*| within the tumor region was graphically assessed by plotting frequency distribution histograms. The percentage of TAMs within the different stiffness categories in treated and untreated animals was compared with a two-way ANOVA followed by Šídák's and Tukey’s multiple comparisons test, respectively. Data are represented as individual values or as mean ± SD. Applied statistical tests are indicated in the figure legends. Multiplicity adjusted p-values are reported where appropriate.

### Supplementary Information

Supplementary Figures.

## Data Availability

The raw numbers for graphs and charts for Figs. 1C–E, 2, 3B,D,F,H, 4A,B,E and 5C are available in the Supplementary source data file. Additional data are available from the authors upon reasonable request.
